# Psychological Symptomatology in Informal Caregivers of Persons with Dementia: Influences on Health-Related Quality of Life

**DOI:** 10.3390/ijerph17031078

**Published:** 2020-02-08

**Authors:** Miguel Madruga, Margarita Gozalo, Josué Prieto, José Carmelo Adsuar, Narcis Gusi

**Affiliations:** 1 Faculty of Sport Sciences, University of Extremadura, 10071 Cáceres, Spain; mgozalo@unex.es (M.G.); jadssal@unex.es (J.C.A.); ngusi@unex.es (N.G.); 2 University School of Education and Tourism, University of Salamanca, 05003 Avila, Spain; josueprieto@usal.es; 3 CIBER of Frailty and Healthy Aging (CIBERFES), 28029 Madrid, Spain; 4 International Institute for Innovation in Aging, 10071 Caceres, Spain

**Keywords:** informal caregivers, Alzheimer’s disease, psychological symptomatology, quality of life, discomfort

## Abstract

Informal caregivers of persons with dementia often report high levels of anxiety, depression and burden. Nonetheless, other less evaluated psychological symptoms might also influence their health-related quality of life (HRQoL). The aim of this study was to comprehensively analyse other psychological symptoms and their influence on the health-related quality of life of informal caregivers. Fifty-four informal women caregivers and fifty-six women non-caregivers were recruited to participate in the study. Psychological symptoms were assessed using the Symptom Check-List-90-Revised (SCL-90-R) questionnaire and the HRQoL with the EuroQoL-Five Dimensions and Three Levels (EurQoL-5D-3L) questionnaire. Significant between-group differences were found in the majority of scales in the SCL-90-R questionnaire (*p* < 0.01) and caregivers also reported a worse HRQoL than non-caregivers (*p* < 0.05). Several psychological symptoms such as obsession-compulsive (β = 0.47), hostility (β = 0.59), and somatization (β = −0.49) had a significant impact on caregivers’ HRQoL (*R*^2^ explained between 0.17 and 0.30 of the variance). Caregivers are at a higher risk of suffering other psychological symptoms and show a moderate–high level of psychiatric morbidity, which therefore explains the poorer HRQoL outcomes. Supporting interventions should be provided to mitigate these psychological symptoms in order to improve their general distress and HRQoL.

## 1. Introduction

At the present time, dementia continues to be one of the most frequent causes of dependence in the elderly, and it is one of the most worrying global public health and social care problems facing people today and in the future, posing a challenge for public health systems and policy makers [1].

The prevalence of dementia is estimated to vary between 5.4% and 14.9% in people older than 65, and 7% and 19.2% in those over 70 years of age [2], with an average of around 10% [3]. The ongoing growth in the number of elderly people together with the rise in life expectancy and other sociodemographic factors are contributing to the increasing need for people to take care of patients with dementia (formal and informal caregivers).

The provision of extraordinary care for a person with Alzheimer’s disease or other dementias to develop activities of daily living (ADLs) and instrumental activities of daily living (IADLs) resides in a person who is considered as a caregiver [1]. Sometimes the role of caregiver may be perceived as a satisfying and rewarding task [4,5]; nevertheless, providing continued support to people with dementia entails an enormous short and long term effort because of the irrepressible decline caused by the disease [6]. In addition, the task may prove difficult, requiring caregivers to face stressful and uncontrolled situations as a result of having to deal with a wide range of care conditions, some of which are very complex [7].

The hazardous tasks that caregivers perform every day have profound consequences for their health [8]. In this regard, caregivers of dementia patients in general, and patients with Alzheimer’s disease (AD) in particular, are at a high risk of developing physical and mental problems that affect their health-related quality of life (HRQoL), as evidenced in several studies [9,10,11,12]. It has also been proved that being a long-term informal caregiver has a significant impact on their psychological and physical health and social environment; in particular, caregivers of AD patients are more likely to endure several psychological discomforts. In this regard, the most widely investigated and documented symptoms in this population are anxiety, depression and burden [8,12,13,14,15]. Nevertheless, other research has reported that caregivers also suffer high levels of stress, emotional strain [13,16,17], social isolation [12] and sleep problems [18,19]. They have also reported irritability, hallucinations, delusions, restlessness, disinhibition, appetite alterations, hostility, somatization and abusive behaviour from the patients [20,21]. All this symptomatology has a negative impact on the general psychological status, physical health and quality of life of caregivers [21,22]; nevertheless, the majority of them have been less comprehensively evaluated, particularly AD caregivers, because of the heterogeneity of their profiles, their specific needs, and the failure to use instruments that specifically measure their psychological symptomatology [8].

For this reason, it could be useful to analyse these less evaluated psychological symptoms and, additionally, study the relationship between this psychological symptomatology and health-related quality of life in caregivers of patients with AD, so as to develop appropriate programmes to reduce their psychological discomfort and improve their HRQoL.

This study aimed to evaluate other psychological symptoms and health-related quality of life in women caregivers of AD patients compared to non-caregivers, as well as to analyse the relationship between HRQoL and these symptoms, and to determine what symptoms influence health-related quality of life in this population.

## 2. Materials and Methods

### 2.1. Participants

This investigation used a cross-sectional community-based study design and was conducted on a sample of women informal caregivers and non-caregivers. Participants were recruited in cooperation with the regional associations of relatives of patients with Alzheimer’s disease. Potential participants were identified by the research team and screened for eligibility based on inclusion and exclusion criteria. Inclusion criteria of the study were: (a) being a woman; (b) being an unpaid family member who offers a substantial amount of care for a person with Alzheimer’s disease or other dementias; (c) providing at least 20 h of in-person care per week [10]; (d) being aged 50 years or above. Exclusion criteria for caregivers were having any external support for caregiving (economic or personnel) or patients with more than one caregiver. After sending all those identified an invitation to participate in the study, 63 caregivers asked for more detailed information about the study. Once they were informed about the protocol, 54 eligible persons consented to participate. The non-caregivers were recruited from two urban and two rural primary care centres in the central area of Caceres (Spain). We located the participants by recruiting general practitioners, who in turn informed individuals about the protocol. Seventy non-caregivers asked for more detailed information about the study, and 56 eligible persons consented to participate. Written informed consent was obtained from all participants in the study.

The sample size was calculated by the proportion of caregivers from the Spanish data set of a healthy population [23] for a hypothetical nonparametric analysis using the Mann–Whitney U test comparing two groups with a significance level less than 0.05 and 90% of the power needed for a minimal clinically relevant difference of 0.5 standard deviation SD (z-score). To achieve these significant effects, 87 participants were required. Allowing for 20% dropout, a total number of participants of 110 was finally recruited.

### 2.2. Measures

A number of sociodemographic characteristics were documented using a questionnaire. Health habits (smoking, alcohol intake, and minutes of physical activity) were additionally measured.

The subjective burden of caregivers was assessed with the Spanish version of the Zarit Burden Interview (ZBI), validated and adapted by Martin et al. in 1996. The original version was developed by Zarit, Reever and Bach-Peterson in 1980 [24] and consisted of a 22-item self-administered questionnaire that examines the burden of caregivers according to a scale of 0–88 associated with functional, psychological, behavioural and economic impairments and the home care situation. The Spanish adaptation of the ZBI establishes the following cut-off scores: 22–46, no burden; 47–56 moderate burden; and 57–110, severe burden [25].

The psychological symptomatology of the participants was evaluated with the Symptom Check List (SCL)-90-Revised [26]. It is a 90-item self-report questionnaire aimed at assessing the presence of psychological distress and a range of psychopathological symptoms. The SCL-90-R yields nine clinical subscales of symptoms (somatization, obsessive-compulsive, interpersonal sensitivity, depression, anxiety, hostility, phobic anxiety, paranoid ideation, and psychoticism), and three global distress indexes. Each item is valued by the respondent on a 5-point Likert scale, which ranges from 0 (not at all) to 4 (extremely). The combined answers on the checklist provide scores for different symptoms that reflect their severity. By converting the raw scores into T-scores, it is possible to compare individual values with normative cut-off values. Each item’s score can be interpreted as being below, within, above or definitely above the average scores of the normative sample, thus indicating the presence of severe symptomatology, resulting in being positive for “caseness” [26]. This occurs when a respondent has a global severity index (GSI) greater than or equal to a T score of 63, or if any two primary dimension scores are greater than or equal to a T score of 63 or the 75th percentile. This questionnaire has been validated and translated into many languages, including Spanish. Thus, the Spanish version of the SCL-90-R [27] was used to assess the psychological symptoms experienced by the caregivers in this study.

Health-related quality of life was assessed with the Spanish version of the EuroQoL-5 Dimensions and 3 Levels (EQ-5D-3L) questionnaire [28]. The EQ-5D-3L by EuroQoL Group [29] is a simple and generic HRQoL questionnaire with two principal measurement components. The first is a descriptive system which defines HRQoL in terms of five dimensions (mobility, self-care, usual activities, pain/discomfort and anxiety/depression). Each dimension has three levels of severity (1 = no problems, 2 = moderate problems and 3 = severe problems). The respondents select one level for each dimension. Using a combination of these dimensions, a total of 243 possible health states exists. Each health state has been previously defined using the time trade-off (TTO) method, which defines a person’s health state in terms of a five-figure code. The second part of the EQ-5D-3L is the visual analogue scale (VAS), which consists of a 20 cm vertical line that ranges from a score of 0 to 100, where 0 represents the worst imaginable health state and 100 represents the best imaginable health state.

### 2.3. Procedure

When permission was granted by the University Research Ethics Committee, the researchers in charge of implementing the questionnaires were informed of how to proceed with each person. Thus, each participant was also informed about the purpose of the study, the confidentiality of the data and filled in the required informed consent form. Subsequently, each participant was interviewed individually at home in a session lasting from about forty to fifty minutes. The study was registered with the clinical trial identifier ISCRCTN80414567 and conducted in accordance with the updates of the Declaration of Helsinki.

### 2.4. Statistical Analysis

Descriptive analyses were conducted to draw a clear profile of the characteristics of the study participants. Mean and standard deviation (SD) were used for continuous variables, frequencies, and percentages for categorical variables. To determine possible baseline differences between groups, participants’ characteristics were analysed using independent sample t-tests for continuous variables if normally distributed (determined by the Kolmogorov–Smirnov test and the Lilliefors correction), and Pearson’s chi square test was performed for categorical variables.

Statistical differences between caregivers and non-caregivers were calculated using the Mann–Whitney U test. Correlations between the main study variables and HRQoL were calculated using Pearson’s coefficient with the Bonferroni adjustment. Finally, to understand which psychological symptoms or other caregiver variables were predictors of HRQoL, a stepwise linear regression analysis was also performed, which included the variables that showed statistically significant associations with caregiver HRQoL. Given the number of comparisons, the level of statistical significance for correlations was set at *p* < 0.005, while for other tests and the regression model, it was set at *p* < 0.05. Statistical calculations were performed using SPSS 18.0 (SPSS Inc., Chicago, IL, USA).

## 3. Results

### 3.1. Sociodemographic Characteristics

The sociodemographic characteristics of the participants are presented in Table 1. A total of 110 women aged between 50 and 75 were recruited in the study. Of these, 54 were caregivers and 56 were non-caregivers. Caregivers had an average age of 60.6 *±* 6.6 years, while non-caregivers had an average age of 62.6 *±* 5.6 years. The majority of participants lived in urban areas (72.2%), were married (79.6%), lived with their husbands (77.8%) and had a primary level of education (81.5%). The most common profile of the caregivers participating in this study was that of a daughter caring for an AD patient for more than five years while living with them. Caregivers reported a lower level of smoking (*p* = 0.051) and they were less physically active than non-caregivers (*p* < 0.01). However, the two groups did not differ significantly in other sociodemographic characteristics, namely place of residence, marital status, living with another person, educational level, and alcohol consumption. Results also showed that caregivers showed a mild to severe level of subjective burden according to the parameters established by Martin et al. (1980) [25].

### 3.2. Differences in Health and Psychological Symptoms

Table 2 shows the scores of the nine dimensions and the global severity index from the SCL-90-R reported by the participants. The differences between both study groups were statistically significant for several dimensions including somatization, obsession-compulsive, depression, anxiety, hostility and the global severity index.

Statistically significant differences were also obtained at the level of quality of life as assessed by the EQ-5D-3L questionnaire, specifically in pain/discomfort and in anxiety/depression dimensions (*p* < 0.01) (see Table 3). Caregivers also presented with worse health than non-caregivers and scored lower on the perception of their health status assessed by the VAS (*p* < 0.01). Additionally, caregivers also declared a worse health status than non-caregivers (*p* < 0.05) as calculated by the TTO technique. However, the two groups did not differ significantly in other dimensions such as mobility, self-care and usual activities.

### 3.3. Relationship between the HRQoL and other variables in caregivers

As shown in Table 4, several dimensions from the SCL-90-R showed significant correlations with HRQoL, including somatization, obsessive-compulsive, interpersonal sensitivity, depression, anxiety, phobic anxiety and psychoticism. Years as a caregiver and overburden were also correlated. On the contrary, no correlations were found for the control group. 

To calculate the predictive models of caregiver HRQoL, factors were selected that were significantly associated with the outcome variable in the bivariate analysis after the Bonferroni adjustment (somatization and obsessive-compulsive) and included in the model. In addition, overburden and other demographic variables were also included in the model (age, years as a caregiver, tobacco and alcohol consumption). The stepwise linear regression analysis showed that somatization and overburden were the main predictive variables of HRQoL, while Aage, years as a caregiver, and tobacco and alcohol behaviours were excluded from the model (see Table 5). Nevertheless, for the self-perceived status of health in caregivers evaluated with the VAS, overburden was the most predictable variable. The R^2^ in model two was 28.1, predicting nearly one-third of the variability in the HRQoL of caregivers.

## 4. Discussion

The main finding of the current study was to identify additional psychological symptoms and other variables besides anxiety and depression that can predict decreasing HRQoL levels in caregivers of patients with Alzheimer’s disease. 

Strong evidence of relationships between psychological impairments caused by caregiving duties and health consequences for caregivers has been previously documented, essentially subjective burden and depression [30]. In this regard, several factors have been considered as mediators of this relationship, including gender, time spent caring, stage of disease, neuropsychiatric symptoms, functional dependency and cognitive impairment of the patient, older age, level of education, socioeconomical status, and social support, among others [30,31]. However, all these factors are further modulated by individual differences among caregivers [32], which are less modifiable. Previous studies also noted that several factors such as worries about the future and the progression of illness, quality of life of patients, age, andsubjective burden have been associated with a decreasing HRQoL in caregivers [13,33]. Nevertheless, there might be other factors that have been less investigated to date that may help to better explain the relationship between psychological symptoms and HRQoL for these informal caregivers [32]. According to recent research, symptoms such as hostility, obsessive-compulsive, and paranoid ideation, among others, have been somewhat documented in studies of spousal caregivers [34,35,36], but this psychological symptomatology is less reported in other populations who do not have the responsibility of looking after their relatives every day. For this reason, our study also compared these psychological symptoms between caregivers and non-caregivers, finding that caregivers reported higher levels of general distress than matched controls [32]. This symptomatology could be derived from patient behaviour problems, social desirability, or the need to provide increasing levels of daily living assistance [20,34]. In fact, the caregivers in our study spent more than twenty hours per week taking care of their patients, possibly giving the impression of permanent care. In particular, somatization episodes can be due to the ailments derived from physical problems such as back pain, pain in the knees, body pain or fatigue that have been reported in previous studies with caregivers of patients with AD [11,12,14,22].

Thus, to provide a better understanding of caregivers’ psychological status and the influence on their HRQoL, our study analysed the relationships between these psychological symptoms and quality of life, finding that most of them were significantly related to the HRQoL of caregivers. The studies developed by Franco et al. [21] and Abreu et al. [36] previously referred to some of these symptoms, however they did not analyse their influence on the HRQoL of caregivers. In this regard, our study found several psychological symptoms evaluated with the SCL-90-R that may have a significant influence on health status and health-related quality of life in caregivers of patients with Alzheimer’s disease. As the outcomes reflected, high scores of somatization have a negative impact on general distress which may reduce their level of health-related quality of life.

Other findings reported in the present study are consistent with previous research; we also found that caregivers had a poorer HRQoL than non-caregivers [10,11,12,22,32,33] and experience more pain/discomfort and anger/hostility [37]. This may be due to their daily caring tasks, the functional disabilities of the patients, and the duties involved in patient supervision [38]. In addition, the EQ-5D-3L has been proven to be a feasible tool for measuring HRQoL in specific populations groups [39]; however, it has been less used to analyse the relationship between HRQoL and other caregiver factors such as psychological symptoms [32].

To our knowledge, this is the first study in which the psychological symptoms of caregivers and non-caregivers of Alzheimer’s disease patients have been compared and analysed using the SCL-90-R questionnaire. Although this questionnaire has not yet been fully explored as a screening method in every potential population, it shows enough specificity and sensitivity to define a positive psychiatric case for different populations [40,41,42]. A person will be assigned as a case by being above (or equal to) a cut-off of 63 in the t-transformed SCL-90-R total score (global severity index) or by being within at least its subscales [41,43]. In the present study, caregivers scored between the 65–70th percentile for the GSI (49.7 ± 10.2), somatization (50.2 ± 9.8) and obsessive-compulsive (51.1 ± 10.1) dimensions. Therefore, we conclude that these caregivers were relatively close to the cut-off for being considered as a positive psychiatric case, and thus showed a high level of psychiatric morbidity [40,42].

The present research has some limitations that should be highlighted. The size of the sample was not very large, which restricts the generalisation of the results as normative values for AD caregivers; therefore, the results have to be interpreted with caution. In addition, as the study was cross-sectional in design, we were not able to assess the variables over time or identify whether their ratings were due to the caregiver role or another factor. Longitudinal research should be conducted to confirm this aspect. Other variables could have been included in our analysis such as the patient’s psychological and physical health, the stage of the Alzheimer’s disease in the care recipient, economic support, social costs, etc.; however, in accordance with Pinquart and Sorensen [14], our study was focused on the variables that could be meaningfully assessed in both groups.

## 5. Conclusions

The findings of this study provide a greater understanding of the psychological symptomatology that caregivers suffer in their daily lives, which is more severe than in non-caregivers. In this regard, in addition to depression, anxiety and burden depicted in previous research, there are other less evaluated psychological symptoms that may significantly influence the health-related quality of life of informal caregivers. Therefore, the assessment of these psychological symptoms might be added to the long list of caregivers’ health problems in order to develop appropriate and tailored programmes to improve their mental health and HRQoL (educational and physical activity-based programmes, supporting and counselling, etc.). Additionally, these symptoms should possibly even be included in assessment protocols to analyse the health status of patients with AD [21] in order to contribute to the development of combined patient–caregiver treatments or interventions.

Future investigations are needed to evaluate the best strategies among those already described to improve caregivers’ psychological symptomatology and HRQoL considering these specific symptoms.

## Figures and Tables

**Table 1:** Sociodemographic, health, and caregiving characteristics of participants.

	Caregivers (*n* = 54)	Non-Caregivers (*n* = 56)	*p* *
*Sociodemographic characteristics*			
Age in years, mean (SD)	60.6 (6.6)	62.6 (5.6)	0.091
Living in urban areas, *n* (%)	39 (72.2)	39 (69.6)	0.766
Marital status married, *n* (%)	43 (79.6)	45 (80.4)	0.167
Living with husband, *n* (%)	42 (77.8)	43 (76.8)	0.974
Primary school education, *n* (%)	44 (81.5)	47 (83.9)	0.237
*Health characteristics*			
Non-smoker, *n* (%)	47 (87.0)	56 (100)	0.051
Non-alcohol consumer, *n* (%)	38 (70.4)	50 (89.3)	0.101
Physically inactive ^a^, *n* (%)	40 (74.2)	17 (30.4)	<0.001
*Caregiving characteristics*			
Dementia: Alzheimer’s, *n* (%)	44 (81.5)		
Relative: daughter, *n* (%)	32 (59.3)		
Living with patient, *n* (%)	40 (74.1)		
Years since diagnosis of dementia, mean (SD)	6.02 (3.96)		
Years as a caregiver, mean (SD)	5.49 (3.32)		
Overburden in caregivers, mean (SD)	55.81 (13.9)		

* *p*-values of analysis from Student’s t test or Chi Square. ^a^ Physically inactive includes respondents who never walk at least 30 min·d^−1^. SD = standard deviation.

**Table 2:** Comparison of psychological symptoms and global severity index (from the Symptom Check-List-90-Revised (SCL-90-R)) between participants.

	Group	Mean (SD)	95% CI	*p ^a^*
SCL-90-R				
Somatization	Caregiver	0.97 (0.86)	[0.73–1.21]	0.001 **
	Non-caregiver	0.37 (0.24)	[0.31–0.44]	
Obsessive-compulsive	Caregiver	0.98 (0.93)	[0.72–1.23]	0.037 *
	Non-caregiver	0.44 (0.26)	[0.37–0.51]	
Interpersonal sensitivity	Caregiver	0.64 (0.67)	[0.45–0.82]	0.274
	Non-caregiver	0.34 (0.24)	[0.28–0.41]	
Depression	Caregiver	0.99 (0.79)	[0.77–1.21]	0.001 **
	Non-caregiver	0.30 (0.23)	[0.23–0.36]	
Anxiety	Caregiver	0.80 (0.84)	[0.57–1.03]	0.001 **
	Non-caregiver	0.25 (0.19)	[0.19–0.30]	
Hostility	Caregiver	0.63 (0.72)	[0.44–0.83]	0.001 **
	Non-caregiver	0.17 (0.22)	[0.11–0.23]	
Phobic anxiety	Caregiver	0.53 (0.75)	[0.32–0.73]	0.144
	Non-caregiver	0.19 (0.24)	[0.13–0.26]	
Paranoid ideation	Caregiver	0.68 (0.73)	[0.48–0.88]	0.870
	Non-caregiver	0.47 (0.29)	[0.39–0.55]	
Psychoticism	Caregiver	0.40 (0.51)	[0.26–0.54]	0.311
	Non-caregiver	0.23 (0.22)	[0.17–0.29]	
Global severity index	Caregiver	0.78 (0.67)	[0.59–0.96]	0.001 **
	Non-caregiver	0.32 (0.13)	[0.28–0.35]	

^a^*p*-values calculated by Mann–Whitney U test. * *p* < 0.05; ** *p* < 0.01.

**Table 3:** Comparison of health-related quality of life (from EuroQol-Five Dimensions-Three Levels (EQ-5D-3L)) between participants.

		Caregivers *n* = 54	Non-Caregivers *n* = 56	*p*
EQ-5D-3L		*n* (%)	*n* (%)	
Mobility ^a^	No problems	48 (88.9)	48 (85.7)	0.619
	Some problems	6 (11.1)	8 (14.3)	
	Extreme problems	0	0	
Self-care ^a^	No problems	52 (96.3)	55 (98.2)	0.539
	Some problems	2 (3.7)	1 (1.8)	
	Extreme problems	0	0	
Usual activities ^a^	No problems	47 (87.0)	54 (96.4)	0.074
	Some problems	7 (13.0)	2 (3.6)	
	Extreme problems	0	0	
Pain/ Discomfort ^a^	No problems	11 (20.4)	34 (60.7)	0.001 **
	Some problems	35 (64.8)	17 (30.4)	
	Extreme problems	8 (14.8)	5 (8.9)	
Anxiety/ Depression ^a^	No problems	25 (46.3)	41 (73.2)	0.007 **
	Some problems	26 (48.1)	12 (21.4)	
	Extreme problems	3 (5.6)	3 (5.4)	
HRQoL (VAS) ^b^; mean (SD)		65.93 (12.96)	80.09 (15.88)	0.001 **
HRQoL (by TTO) ^b^; mean (SD)		0.76 (0.22)	0.86 (0.19)	0.024 *

^a^ Calculated by chi-Square (*χ2*); ^b^ Calculated by Student’s t- test; * *p* < 0.05; ** *p* < 0.01. TTO = time trade-off; VAS = visual analogue scale.

**Table 4:** Bivariate correlations between health-related quality of life and the main study variables.

		Caregivers	
	Mean (SD)	r	*p*
HRQoL (by TTO)	0.76 (0.22)		
SCL-90-R dimensions ^a^			
Somatization	0.97 (0.86)	−0.42 *	0.001
Obsessive-compulsive	0.98 (0.93)	−0.38 *	0.004
Interpersonal sensitivity	0.64 (0.67)	−0.31	0.020
Depression	0.99 (0.79)	−0.29	0.034
Anxiety	0.80 (0.84)	−0.32	0.015
Hostility	0.63 (0.72)	−0.13	0.317
Phobic anxiety	0.53 (0.75)	−0.32	0.016
Paranoid ideation	0.68 (0.73)	−0.22	0.099
Psychoticism	0.40 (0.51)	−0.28	0.038
Years as a caregiver	5.49 (3.32)	−0.27	0.049
Overburden	55.81 (13.9)	−0.36	0.007

^a^ Calculated using Pearson’s correlation; * *p* < 0.05 (adjusted using the Bonferroni test < 0.005).

**Table 5:** Summary of the stepwise regression analysis of health-related quality of life and associated variables.

Dependent Variables	Predictor Variables	*F*	*B*	*β*	*t*	*p*	R^2^
HRQoL ^a^	Somatization (Model 1)	11.28	−0.11	−0.42	−3.35	0.001 **	0.178
	Somatization Overburden (Model 2)	9.94	−0.10 −0.05	−0.39 −0.32	−3.26 −2.69	0.002 ** 0.010 *	0.281
Health status ^b^	Overburden	14.55	−0.43	−0.46	−3.85	<0.01 **	0.219

Note: *F* value (ANOVA); B = unstandardized βeta; β = standardized βeta coefficient; *t* = *t* test statistics; *R*^2^ = coefficient of determination. ^a^ health-related quality of life (by TTO); ^b^ health-related quality of life (by VAS); * *p* < 0.05; ** *p* < 0.01.

## References

[1] Alzheimer Disease International (2016). World Alzheimer Report 2016. Improving healthcare for People Living with Dementia: Coverage, Quality and Costs Now and in the Future.

[2] Garcés M. (2016). Estudio Sobre las Enfermedades Neurodegenerativas en España y su Impacto Económico y Social.

[3] De Pedro-Cuesta J., Virues-Ortega J., Vega S., Seijo-Martinez M., Saz P., Rodriguez F., Rodriguez-Laso A., Rene R., de las Heras S.P., Mateos R. (2009). Prevalence of dementia and major dementia subtypes in Spanish populations: A reanalysis of dementia prevalence surveys, 1990–2008. BMC Neurol..

[4] Ploeg J., Markle-Reid M., Valaitis R., McAiney C., Duggleby W., Bartholomew A., Sherifali D. (2017). Web-Based Interventions to Improve Mental Health, General Caregiving Outcomes, and General Health for Informal Caregivers of Adults With Chronic Conditions Living in the Community: Rapid Evidence Review. J. Med. Internet Res..

[5] Ribeiro O., Brandao D., Oliveira A.F., Teixeira L., Paul C. (2019). Positive aspects of care in informal caregivers of community dwelling dementia patients. J. Psychiatr. Ment. Health Nurs..

[6] Vandepitte S., Van Den Noortgate N., Putman K., Verhaeghe S., Annemans L. (2016). Effectiveness and cost-effectiveness of an in-home respite care program in supporting informal caregivers of people with dementia: Design of a comparative study. BMC Geriatr..

[7] Van’t Leven N., Prick A.E., Groenewoud J.G., Roelofs P.D., de Lange J., Pot A.M. (2013). Dyadic interventions for community-dwelling people with dementia and their family caregivers: A systematic review. Int. Psychogeriatr..

[8] Pérez-Fuentes M., Gázquez J., Ruiz M., Molero M. (2017). Inventory of overburden in Alzheimer’s patient family caregiver with no specialized training. Int. J. Clin. Health Psychol..

[9] Flores N., Jenaro C., Moro L., Tomsa R. (2014). Salud y calidad de vida de cuidadores familiares y profesionales de personas mayores dependientes: Estudio comparativo. Eur. J. Investig. Health Psychol. Educ..

[10] Badia X., Lara N., Roset M. (2004). Quality of life, time commitment and burden perceived by the principal informal caregiver of Alzheimer’s patients. Rev. Aten. Primaria.

[11] Gusi N., Prieto J., Madruga M., Adsuar J., González-Guerrero J., García -Domínguez J. (2009). Health-related quality of life and fitness differences between family caregivers of patient with dementia and non-caregivers. Med. Sci. Sport Exerc..

[12] Ho S.C., Chan A., Woo J., Chong P., Sham A. (2009). Impact of caregiving on health and quality of life: A comparative population-based study of caregivers for elderly persons and noncaregivers. J. Gerontol. Ser. A Biol. Sci. Med. Sci..

[13] Chiao C.Y., Wu H.S., Hsiao C.Y. (2015). Caregiver burden for informal caregivers of patients with dementia: A systematic review. Int. Nurs. Rev..

[14] Pinquart M., Sorensen S. (2003). Differences between caregivers and noncaregivers in psychological health and physical health: A meta-analysis. Psychol. Aging.

[15] Salazar A., Murcia L., Solano J. (2016). Evaluación e intervención de la sobrecarga del cuidador informal de adultos mayores dependientes: Revisión de artículos publicados entre 1997–2014. Arch. Med..

[16] Farran C.J., Paun O., Cothran F., Etkin C.D., Rajan K.B., Eisenstein A., Navaie M. (2016). Impact of an Individualized Physical Activity Intervention on Improving Mental Health Outcomes in Family Caregivers of Persons with Dementia: A Randomized Controlled Trial. AIMS Med. Sci..

[17] Marquez-Gonzalez M., Losada-Baltar A., Izal M., Perez-Rojo G., Montorio I. (2007). Modification of dysfunctional thoughts about caregiving in dementia family caregivers: Description and outcomes of an Intervention Program. Aging Ment. Health.

[18] Losada-Baltar A., Izal M., Montorio I., Márquez-González M., Pérez-Rojo G. (2004). Eficacia diferencial de dos intervenciones psicoeducativas para cuidadores de familiares con demencia. Rev. Neurol..

[19] Thomas P., Hazif-Thomas C., Pareault M., Vieban F., Clément J.P. (2010). Sleep disturbances in home caregivers of persons with dementia. Encephale.

[20] Feast A., Orrell M., Russell I., Charlesworth G., Moniz-Cook E. (2017). The contribution of caregiver psychosocial factors to distress associated with behavioural and psychological symptoms in dementia. Int. J. Geriatr. Psychiatry.

[21] Franco C., Sola M., Justo E. (2010). Reducing psychological discomfort and overload in Alzheimer’s family caregivers through a mindfulness meditation program. Rev. Esp. Geriatr. Gerontol..

[22] Garzón-Maldonado F., Gutiérrez-Bedmar M., García-Casares N., Pérez-Errázquin F., Gallardo-Tur A., Martínez-Valle M. (2016). Calidad de vida relacionada con la salud en cuidadores de pacientes con enfermedad de Alzheimer. Neurologia.

[23] Rodríguez F., Gusi N., Valenzuela A., Nácher S., Nogués J., Marina M. (1998). Evaluation of health-related fitness in adults (I): Background and protocols of the AFISAL-INEFC battery. Apunt. Educ. Física Deportes.

[24] Zarit S.H., Reever K.E., Bach-Peterson J. (1980). Relatives of the impaired elderly: Correlates of feelings of burden. Gerontology.

[25] Martin M., Salvado I., Nadal S., Miji L., Rico J., Lanz P., Taussing M. (1996). Adaptation to our mean of caregiver burden scale of Zarit. Rev. Gerontol..

[26] Derogatis L. (1977). SCL-90-R, Administration, Scoring and Procedures Manual I for the Revised Version of the SCL-90.

[27] Gonzalez de Rivera J., Derogatis L., De las Cuevas C., Gracia R., Rodríguez-Pulido F., Henry-Benítez M., Monterrey A. (1989). The Spanish Version of the SCL-90-R. Normative Data in the General Population.

[28] Badia X., Roset M., Monserrat S., Herdman M., Segura A. (1999). The Spanish version of EuroQol: Description and uses. Med. Clin..

[29] EuroQol Group (1990). EuroQol-a new facility for the measurement of health-related quality of life. Health Policy.

[30] Viñas-Díez V., Conde-Sala J.L., Turró-Garriga O., Gascón-Bayarri J., RReñé-Ramírez R. (2019). Síntomas depresivos y sobrecarga en los familiares cuidadores en la enfermedad de Alzheimer: Un modelo de ecuaciones estructurales. Rev. Neurol..

[31] González-de Paz L., Real J., Borrás-Santos A., Martínez-Sánchez J.M., Rodrigo-Baños V., Dolores Navarro-Rubio M. (2016). Associations between informal care, disease, and risk factors: A Spanish country-wide population-based study. J. Public Health Policy.

[32] Del Rio Lozano M., Garcia-Calvente M.D.M., Calle-Romero J., Machon-Sobrado M., Larranaga-Padilla I. (2017). Health-related quality of life in Spanish informal caregivers: Gender differences and support received. Qual. Life Res. Int. J. Qual. Life Asp. Treat. Care Rehabil..

[33] Dawood S. (2016). Caregiver Burden, Quality of Life and Vulnerability towards Psychopathology in Caregivers of Patients with Dementia/Alzheimer’s Disease. J. Coll. Physicians Surg. Pak..

[34] Zghal M., Robbena L., El Ghali F., Ben Ghzaeil I., Rafrafi R. (2016). Hostility and emotional load of Tunisian caregivers of Alzheimer patients and its impact on the patient. Eur. Psychiatry.

[35] Ploeg J., Matthew-Maich N., Fraser K., Dufour S., McAiney C., Kaasalainen S., Markle-Reid M., Upshur R., Cleghorn L., Emili A. (2017). Managing multiple chronic conditions in the community: A Canadian qualitative study of the experiences of older adults, family caregivers and healthcare providers. BMC Geriatr..

[36] Abreu W., Rodrigues T., Sequeira C., Pires R., Sanhudo A. (2018). The experience of psychological distress in family caregivers of people with dementia: A cross-sectional study. Perspect. Psychiatr. Care.

[37] Dew M.A., Goycoolea J.M., Harris R.C., Lee A., Zomak R., Dunbar-Jacob J., Rotondi A., Griffith B.P., Kormos R.L. (2004). An internet-based intervention to improve psychosocial outcomes in heart transplant recipients and family caregivers: Development and evaluation. J. Heart Lung Transpl..

[38] Schulz R., Sherwood P.R. (2008). Physical and mental health effects of family caregiving. Am. J. Nurs..

[39] Wanden-Berghe C., Nolasco A., Planas M., Sanz-Valero J., Rodriguez T., Cuerda C., Guardiola R., Castello-Botia I., Grupo N.-S. (2008). Health-related quality of life according to the main caregiver in patients with home nutritional support. Med. Clin..

[40] Mueller M., Riecher A., Kammermann J., Stieglitz R.D., Stettbacher A., Vetter S. (2009). Prediction of caseness for mental pathology in Swiss conscripts: The Self-Screen Prodrome. Mil. Med..

[41] Sanders-Dewey N., Mullins L., Chaney J. (2001). Coping Style, Perceived Uncertainty in Illness, and Distress in Individuals with Parkinson’s Disease and Their Caregivers. Rehabil. Psychol..

[42] Sinatora F., Traverso A., Zanato S., Di Florio N., Porreca A., Tremolada M., Boscolo V., Marzollo A., Mainardi C., Calore E. (2017). Quality of Life and Psychopathology in Adults Who Underwent Hematopoietic Stem Cell Transplantation (HSCT) in Childhood: A Qualitative and Quantitative Analysis. Front Psychol..

[43] Derogatis L. (1994). SCL-90-R: Administration, Scoring and Procedures Manual.

